# Identification and metabolomic characterization of potent anti-MRSA phloroglucinol derivatives from *Dryopteris crassirhizoma* Nakai (Polypodiaceae)

**DOI:** 10.3389/fphar.2022.961087

**Published:** 2022-10-20

**Authors:** Sumana Bhowmick, Manfred Beckmann, Jianying Shen, Luis A. J. Mur

**Affiliations:** ^1^ Department of Neurology, Stanford University School of Medicine, Stanford, CA, United States; ^2^ Institute of Biological, Environmental and Rural Studies, Aberystwyth University, Aberystwyth, United Kingdom; ^3^ Artemisinin Research Center, Institute of Chinese Materia Medica, China Academy of Chinese Medical Sciences, Beijing, China

**Keywords:** natural products, phloroglucinols, *Dryopteris crassirhizoma*, metabolomics, methicillin-resistant *Staphylococcus aureus*

## Abstract

Traditional Chinese medicine (TCM) has been used to treat infectious diseases and could offer potential drug leads. This study evaluates the *in vitro* antimicrobial activities from commercially sourced *Dryopteris crassirhizoma* Nakai (Polypodiaceae) whose authenticity was confirmed by DNA barcoding based on the ribulose bisphosphate carboxylase (*rbcL*) gene. Powdered rhizomes were sequentially extracted using *n-*hexane, dichloromethane, ethyl acetate, and methanol at ambient temperature. The dried extracts at different concentrations were tested for antimicrobial activities against *Escherichia coli*, *Pseudomonas aeruginosa*, *Staphylococcus aureus*, methicillin-resistant *Staphylococcus aureus* (MRSA), and *Mycobacterium smegmatis*. *D. crassirhizoma* extracts exhibited significant antimicrobial activities only against MRSA (minimum inhibitory concentration: 3.125 μg/ml *n-*hexane extract). Activity-led fractionations of *D. crassirhizoma* and characterization by ultra-performance liquid chromatography–tandem mass spectrometry (UPLC-MS/MS) targeted a fraction (A3), with two anti-MRSA phloroglucinol derivatives, flavaspidic acid AB and norflavaspidic acid AB—being greatly enriched in the latter. The impact of A3 on MRSA cells was examined using untargeted metabolomic analysis and compared to that of other established antibiotics (all treatments normalized to MIC_50_ at 6 h). This suggested that norflavaspidic acid AB had distinctive effects, one of which involved targeting bioenergetic transformation, metabolism, and particularly acetyl-CoA, on MRSA cells. No cytotoxicity was observed for the norflavaspidic acid AB-enriched fraction against murine HepG2 cells. This study requires further experimental validation but can have indicated a naturally available compound that could help counter the threat of clinically relevant strains with antibiotic resistance.

## Introduction

The diversity within the plant kingdom leads it to being the source of important bioactive compounds. Likely sources of bioactives can be suggested from Ayurveda (traditional Indian medicine) dating from about 1000 BC and traditional Chinese medicine (TCM) from 1100 BC. The earliest records of the uses of a natural product are from 2600 BC which describes the use of oils from *Cupressus sempervirens* L. (Cupressaceae) (cypress), *Commiphora species* (myrrh), *Glycyrrhiza glabra* L. (Fabaceae) (liquorice), and *Papaver somniferum* L. (Papaveraceae) (poppy juice) to treat cough, colds, parasitic infections, and inflammation (Cragg and Newman, 2005). Examples of how such ancient medicines can serve as sources of what, ultimately, have been marketed as drugs include paclitaxel (Taxol^®^) from *Taxus brevifolia* Nutt. (Taxaceae) for lung, ovarian, and breast cancer (Kaufman et al., 2010), artemisinin from the traditional Chinese plant *Artemisia annua* L. (Asteraceae) to combat multidrug-resistant malaria (Cragg and Newman, 2005; Paul, 2002), and silymarin extracted from the seeds of *Silybum marianum* (L.) Gaertn. (Asteraceae) for the treatment of liver diseases (Shaker et al., 2010). TCM includes over 7,000 plant species that have medicinal uses and is attracting global interest as a source of natural products. However, information regarding their use is often inaccessible to the wider scientific community, and there can be difficulties in product quality if obtained from commercial sources.

In terms of a target for TCM-derived drugs, perhaps helping to address the challenge of antimicrobial resistance (AMR) is one of the most pressing problems. The rise of AMR is recognized as one of the greatest threats to human health with ∼1 million deaths linked to drug-resistant microbes between 2014 and 2016 (O’Neill, 2016). This reflects that after decades marked by the discovery of antibiotics such as sulphonamides, β-lactams, aminoglycoside, and trimethoprim, the period after 1980 is defined as an “antibiotic discovery void” (Silver, 2011). The major factors underlying the rise of clinically relevant AMR organisms include the extensive and often unnecessary use of antimicrobials providing a strong selective pressure, together with the large and globally connected human population allowing bacterial spread (Michael et al., 2014). One dangerous example of AMR bacterial strains is represented by methicillin-resistant *Staphylococcus aureus* (MRSA). MRSA is a major nosocomial pathogen that has emerged from a hospital setting. At one stage, two strain types, (US 300 and US 400) were the primary causes of community-acquired MRSA infections in the United States (McDougal et al., 2003). The MRSA strains are further differentiated based on two types of community-acquired MRSA (CA-MRSA) and hospital-acquired MRSA (HA-MRSA). In general, HA-MRSA strains exhibit high-level resistance to multiple non-β-lactam antimicrobial agents such as quinolones, aminoglycosides, and macrolides. In contrast, CA-MRSA strains are usually susceptible to non-β-lactams but produce various virulence and colonization factors (Diep & Otto, 2008; Dinges et al., 2000; Zetola et al., 2005). Currently, treatments for severe MRSA infections are vancomycin and daptomycin for bacteremia; vancomycin, daptomycin, or linezolid for complicated skin and soft-tissue infections; and vancomycin or linezolid for hospital-associated pneumonia. However, the overuse of vancomycin in hospitals has resulted in vancomycin-resistant MRSA. Linezolid is still used intensively as resistance to it is currently rare (Itani et al., 2010). Daptomycin also remains effective, but its cost is limiting its use (Bounthavong et al., 2011).

*Dryopteris crassirhizoma* Nakai (Polypodiaceae), a perennial herbaceous fern, known as “the king of antivirals,” is widely distributed in Korea, China, and Japan (Gao et al., 2008). The roots, known as “*Gwanjung*” in Korea; “*Guan Zhong*” in China, and “*Oshida*” in Japan, are used in TCM to treat parasitic infestation, hemorrhage, epidemic flu, cold, and cancer (Committee, 1999; Yang et al., 2003; Shinozaki et al., 2008). Powdered and dried rhizomes of various *Dryopteris* ferns have been used as remedies for helminthiasis caused by *Diphyllobothrium latum* (Murakami and Tanaka, 1988). Previously reported phytochemical constituents include triterpene, phloroglucinol, flavonoids, and other phenolic compounds (Chang et al., 2006; Min et al., 1998; Noro et al., 1973; Shiojima et al., 1990).

Herein, we screen *D. crassirhizoma* for antibacterial activities. The most potent activities were identified as phloroglucinol derivatives whose mechanism of action was suggested by metabolomic studies to be linked to a perturbation of glycolysis in MRSA which appeared to significantly deplete acetyl coenzyme A (acetyl-CoA).

## Results

### Bioassay-guided isolation of bioactives from *D*. *crassirhizoma*

Given that the TCM was obtained from commercial sources as dried leaves, we sought to confirm its identity as *D. crassirhizoma.* To authenticate the samples, we employed DNA barcoding based on the *rbcL* (ribulose-bisphosphate carboxylase) gene. The derived sequences (accession number: MN431197) were identical to *D. crassirhizoma* voucher sequences (e.g., KC896537) found on the National Center for Biotechnology Information (NCBI) GenBank database.

Powdered samples were sequentially extracted using *n-*hexane, dichloromethane (DCM), ethyl acetate (EtOAc), and methanol (MeOH). Minimal inhibitory concentrations (MICs) of each extract were then screened against a range of clinically relevant bacterial strains, *E. coli* (E), *P. aeruginosa* (P), *S. aureus* (S), MRSA, and *M. smegmatis* (M) (Table 1). The *n-*hexane extract of *D. crassirhizoma* was active against S (MIC of 3.125 μg/ml) and against MRSA (MIC of 3.125 μg/ml). The DCM extract was active against S (MIC of 25 μg/ml) and against MRSA (MIC of 50 μg/ml).

**TABLE 1:** Antimicrobial screening of crude *D. crassirhizoma*, given as minimum inhibitory concentrations [MICs] µg/mL.

Plant species	Accession number	Part	Solvents	E	P	M	S	MRSA
*D. crassirhizoma*	MN431197	Rhizome	*n-*Hexane	>500	>500	500	3.125	3.125
			DCM	>500	>500	500	25	50
			EtOAc	>500	>500	>500	>500	>500
			MeOH	>500	>500	>500	>500	>500

E, *Escherichia coli* ATCC 25922; P, *Pseudomonas aeruginosa* ATCC 27853; *Mycobacterium smegmatis* NCTC 333; S, *Staphylococcus aureus* ATCC 29213; MRSA, methicillin-resistant *Staphylococcus aureus* USA300.

**FIGURE 1:**
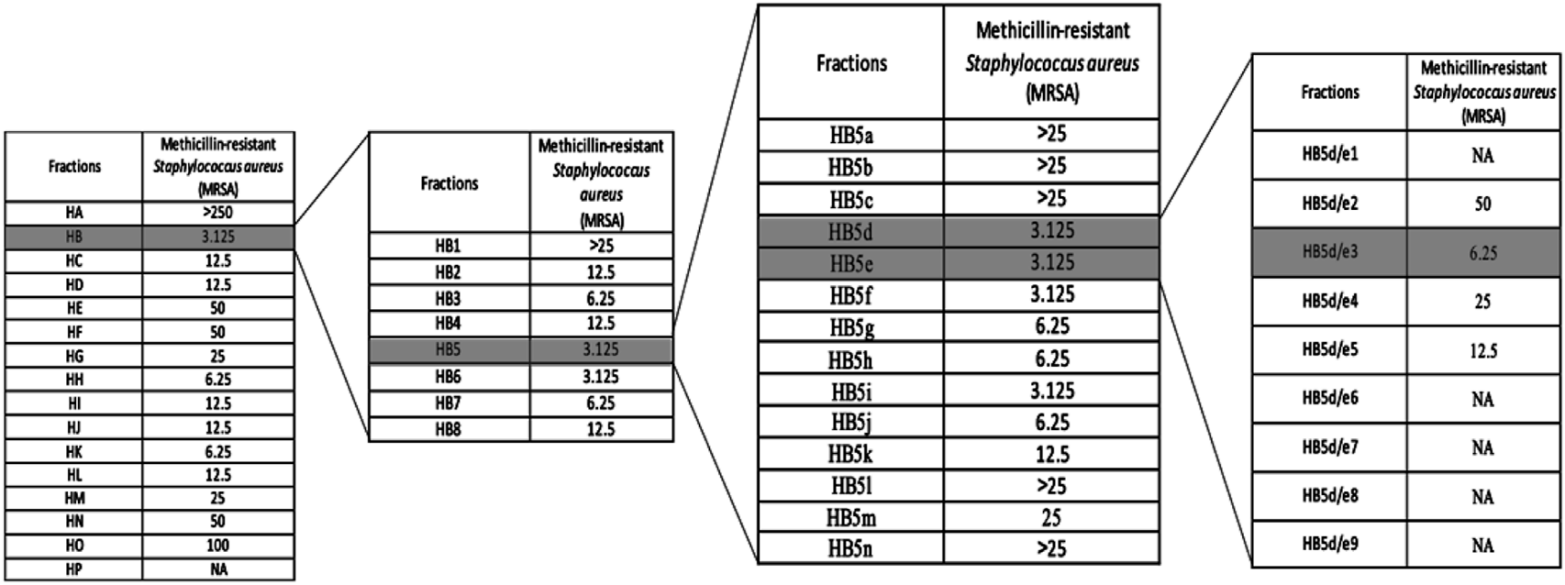
Bioassay-guided purified fractions of n-hexane extracts (MIC µg/mL). Note: NA, not active.

Based on the MIC values against MRSA observed from crude extracts, we focused on the *n-*hexane extract of *D. crassirhizoma* for further purification (Figure 1). After the first round of purification, assays of the resulting fraction indicated as HB displayed the highest anti-microbial activity (MIC of 3.125 μg/ml). Fraction HB was further purified and assessed, and the fraction of HB5 and HB6 (MIC of 3.125 μg/ml, respectively) had the highest activity against MRSA. HB5 was further purified based on its higher quantity and the similarity of its mass ions (*m/z*) with HB6 following UHPLC-MS (Supplementary Figure S1A). Fractions HB5d and HB5e were chosen to be further purified based on their MICs. However, the fractions showed to have very similar biochemical components as detected by UHPLC-MS (Supplementary Figure S1B) and so were combined and further fractionated. HB5d/e3 (MIC, 6.25 μg/ml) was the most active fraction. UHPLC-MS indicated the abundance of *m/z* 405.15417 with a minor level of *m/z* 419.1695 in the positive ionization mode (Supplementary Figure S2). HB5d/e5 (MIC, 12.5 μg/ml) also contained only *m/z* 405.15417 and *m/z* 419.1695, but the intensities were more equal. This implied that *m/z* 405.15417 represented the more potent anti-microbial effect. The fractions underwent several alternative purification techniques, but it was not possible to separate the two analytes. Nuclear magnetic resonance (NMR) was attempted with these fractions, but it was not possible to obtain satisfactory results to confirm the identity of the analytes.

**FIGURE 2:**
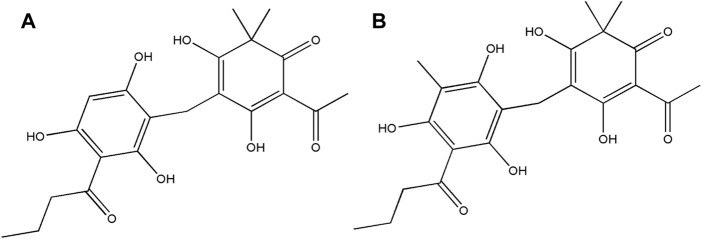
Structures of the bioactive natural products isolated from *D. crassirhizoma*. **(A)** Norflavaspidic acid AB and **(B)** flavaspidic acid.

Thus, the components in HB5d/e3 were characterized and identified via UHPLC-MS coupled with MS^2^/MS^3^ fragmentation based on retention time, accurate mass, elemental composition, and multiple-stage mass data (Table 2, Supplementary Figures S3, S4). This suggested that the two compounds were phloroglucinol derivatives, and when compared with the literature, these were identified as norflavaspidic acid AB (*m/z* 405.15417) and flavaspidic acid AB (Ren et al., 2016) (Figure 2).

**TABLE 2:** Identification of antimicrobial metabolites from *D. crassirhizoma* using UHPLC-MS/MS.

Compound name	t_R_, min	Observed mass	ESI-MS^n^ data (relative intensity %)
Norflavaspidic acid AB	6.64	405.15417	MS^2^ [405]: 197 (100), MS^3^ [405→197]: 179 (100), 155 (19), 151(8),113 (7)
Flavaspidic acid AB	6.94	419.16959	MS^2^ [419]: 223 (9), 211 (61), 197 (100)
			MS^3^ [419→197]: 179 (100), 155 (18), 151 (8), 113 (7)

Given the potent anti-microbial activity of the phloroglucinol derivatives, the cytotoxicity of HB5d/e3 (for simplicity, designed A3) enriched in norflavaspidic acid AB was assessed in HepG2 cell cultures. Screening indicated that the fractions did not show any cytotoxic activities CC_50_ > 100 μM (>40.4 μg/ml). The selectivity index (SI) (CC_50_/MIC) of A3 for HepG2 evaluated in relation to its very potent antibacterial activity against MRSA was >13, which was favorable for further development.

### Untargeted metabolomics suggested the activities of the phloroglucinol derivatives

To suggest the defining possible mode of action for A3 on MRSA, a metabolomic approach was employed. We also aimed to compare A3 effects with established antibiotics with different known cellular targets, namely, chloramphenicol (CH), gentamycin (G), levofloxacin (L), nalidixic acid (N), streptomycin (S), and vancomycin (V). To standardize the antimicrobial effects of each biochemical, the concentrations used in each case were sufficient to inhibit MRSA growth by 50% at 6 h (MIC_50_) (Table 3, Supplementary Figure S5).

**TABLE 3:** Standardization of antibiotic concentrations based on MRSA growth suppression (50%) at different time points of culture.

Compound	MIC_50_ 6h (µg/ml)
A3 (Hb5d/e3)	0.49
L	0.49
S	0.166
V	0.33
CH	1.95
N	7.8125
G	0.49

**FIGURE 3:**
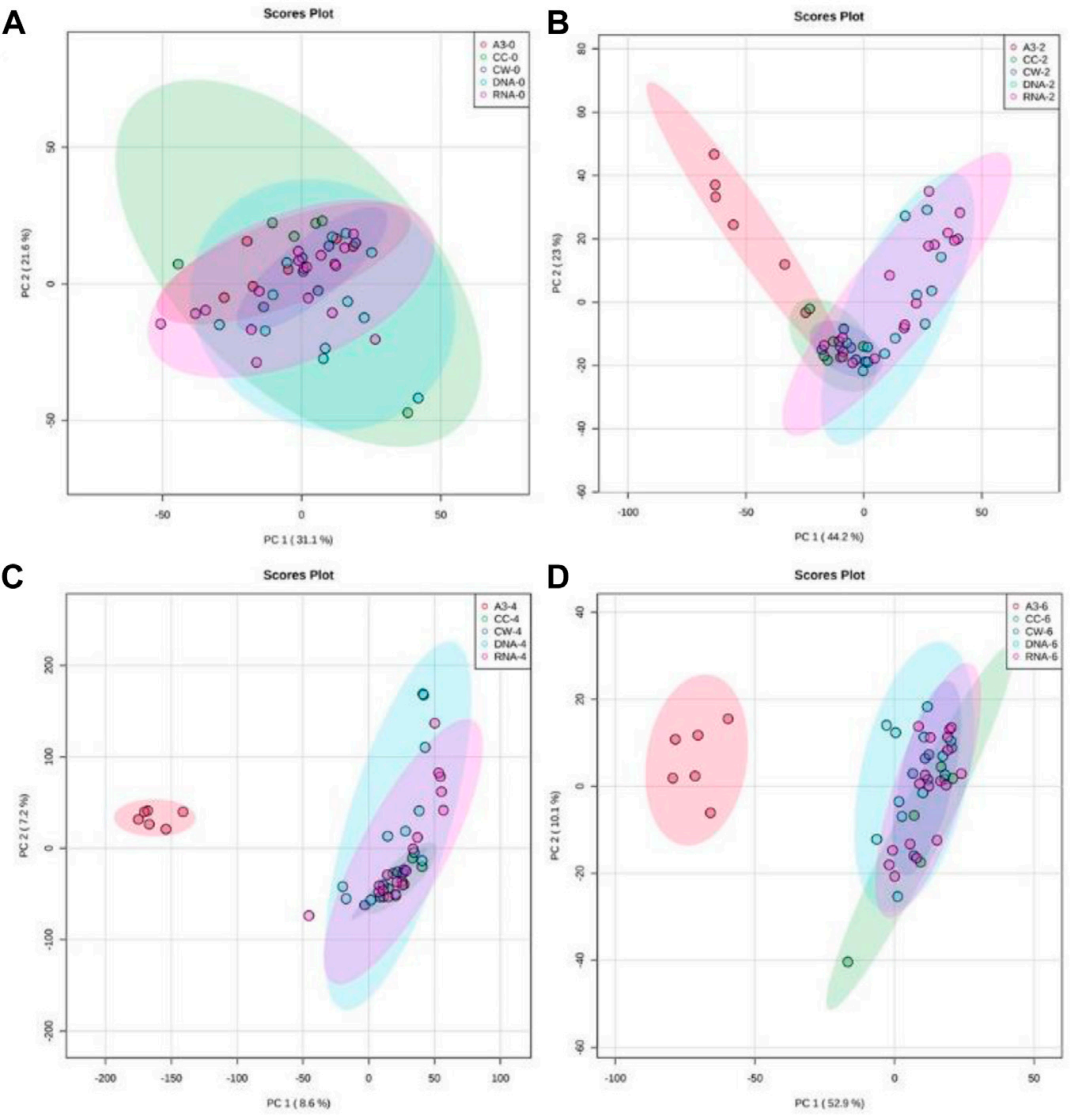
Principal component analysis (PCA) of antibiotic-treated MRSA metabolomes. PCA score plots (95% confidence interval is shown as paler colored ellipses) of normalized m/z intensities of metabolites extracted as detected by flow-infusion electrospray high-resolution mass spectrometry (FIE-HRMS). from MRSA treated with A3 (Hb5d/e3) and compared to control bacteria (CC) and to bacteria treated with antibiotics with a similar mechanism of action, grouped into those with activity on cell wall (vancomycin, CW), on DNA (Levofloxacin and Nalidixic acid) and on RNA (Chloramphenicol, Gentamycin, and Streptomycin) for both positive and negative mode for time points **(A)** 0, **(B)** 2, **(C)** 4 and **(D)** 6 h.

Flow-infusion electrospray ionization high-resolution mass spectrometry (FIE-HRMS) was used to profile extracted metabolites derived from MRSA treated with A3 or CH, G, L, N, S, V, and also untreated control MRSA cells at various time points (Figure 3).

In Figure 3, unsupervised PCA was used to compare metabolomic variation in MRSA treated with A3 and the different established antibiotics at discrete time points. To ease comparison, the established antibiotics were designated according to their modes of action. Thus, vancomycin was designated as targeting the cell wall (CW), levofloxacin and nalidixic acid targeting the DNA structure (DNA), and chloramphenicol, gentamycin, and streptomycin targeting RNA within the ribosome (rRNA). Immediately after treatment, the MRSA metabolomes were identical indicating that the simple addition of A3/antibiotics had no major impact on the derived data (Figure 3A). However, within 2 h of treatment with A3 resulted in a shift in the metabolome so that it was distinct from the changes in MRSA to established antibiotics (Figure 3B). It was notable that the metabolomes of the established antibiotics clustered together and were therefore similar, irrespective of their different modes of action. This co-clustering of the MRSA metabolomes when treated with established antibiotics was also seen at 4 h (Figure 3B) and 6 h (Figure 3D). This did not reflect the MRSA cells being all dead, as the concentrations of antibiotics used only suppressed the bacterial growth by 50% at 6 h. However, this could indicate that the MRSA metabolomes were reflecting a common stress response by the bacteria.

**FIGURE 4:**
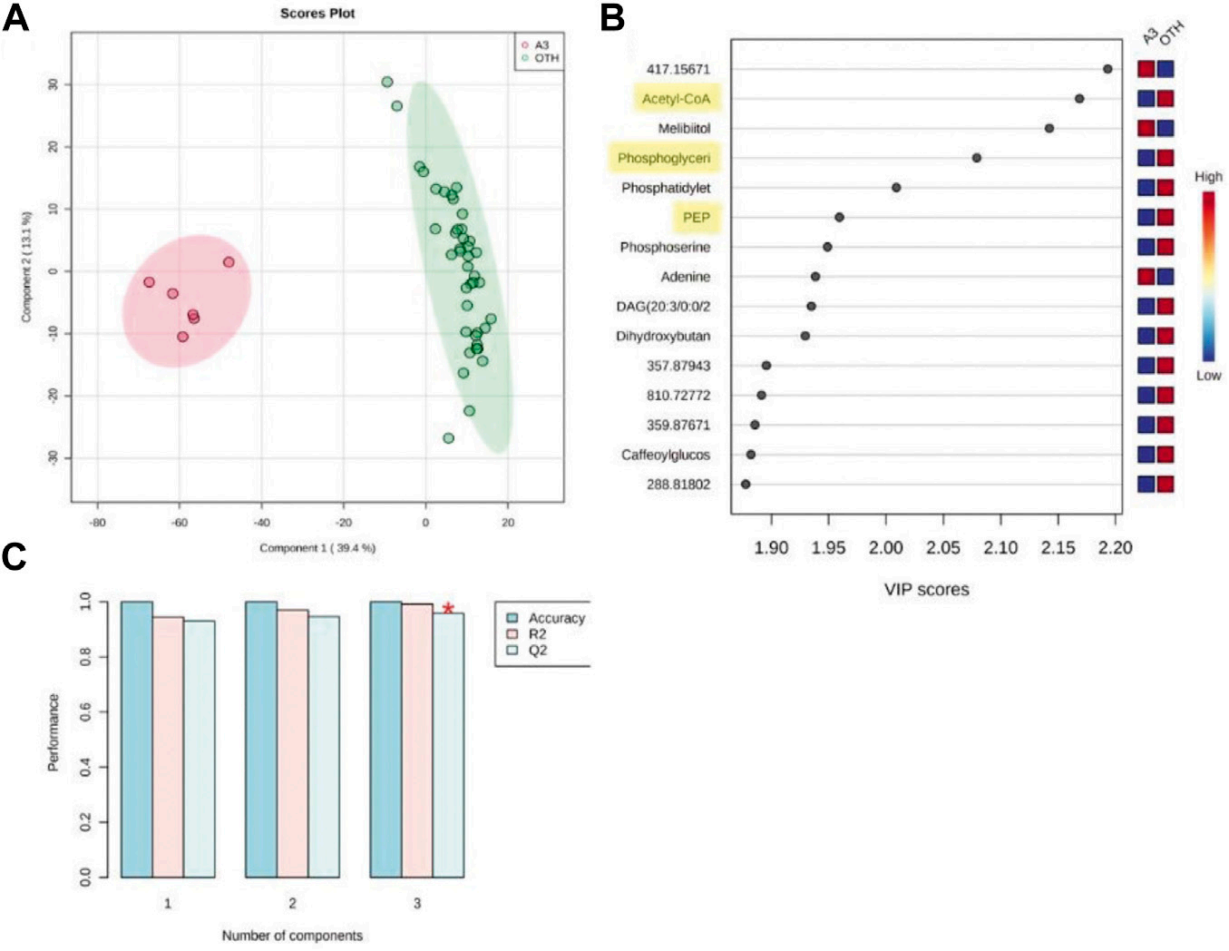
Partial least squared discriminant analysis (PLS-DA) of antibiotic-treated MRSA metabolomes. **(A)** PLS-DA score plots (95% confidence interval illustrated show as paler colored ellipses) of normalized m/z intensities of metabolites extracted as detected by flow-infusion electrospray high-resolution mass spectrometry (FIE-HRMS) from MRSA treated with A3 (Hb5d/e3) and compared to all other (“OTH”) treatments of vancomycin, levofloxacin, and nalidixic acid and chloramphenicol, gentamycin, and streptomycin for both positive and negative modes for time points. **(B)** Validation of the PLS-DA model based on leave-one-out cross-validation (LOOCV) showing the high accuracy of the model, R2, the goodness of fit, and Q2, the goodness of prediction. **(C)** Variable importance in projection (VIP) scores for the PLS-DA. The relative levels of *m/z* with A3 or OTH treatments are indicated. Metabolites involved in glycolysis/gluconeogenesis are highlighted in yellow.

Given that experiment was standardized to a 50% reduction in MRSA growth at 6 h, further analyses were concentrated at that time point. Partial least squares-discriminant analysis (PLS-DA) considered the changes in the MRSA metabolomes at 6 h with A3 compared to all the other (“OTH”) established antibiotics as they appeared to have similar effects on MRSA (Figure 4). PLS-DA biplots reiterated the distinctive effects of A3 on the MRSA metabolome at 6 h (Figure 4A) which did not arise from overfitting given the high accuracies indicated when the model was assessed based on LOOCV (leave-one-out cross-validation) (Figure 4B). The major sources of variation (*m/z*) between A3 and the OTH group were identified by ANOVA (*p =* 0.001) corrected for FDR. They were also assessed based on variable importance in projection (VIP) scores from the PLS-DA model (Figure 4C). The ANOVA outputs were used to tentatively identify the metabolites, using the *mummichog* algorithm in MetaboAnalyst 4.0 (Li et al., 2013). Where there was overlap with the VIPS results, the *m/z* was labeled with the corresponding metabolite. This double approach ensured that only the most important metabolomic changes were targeted. Figure 4C suggests that treatment with A3 resulted in a loss in acetyl-CoA, phosphoglyceric acid, and phosphoenolpyruvate (PEP). This indicated that the levels of glycolysis intermediates—such as phosphoenolpyruvate (PEP) and 3-phosphoglycerate (3-PG)—were greatly decreased in cells treated with A3 (Supplementary Figure S6). This shift in the glycolysis/gluconeogenesis pathway did not arise from a shift toward the production of lactate or to the citrate cycle since these were not targeted as being affected in our assessments. However, the dramatic loss in acetyl-CoA observed could undoubtedly affect the production of citrate and therefore the function of the TCA cycle.

**FIGURE 5:**
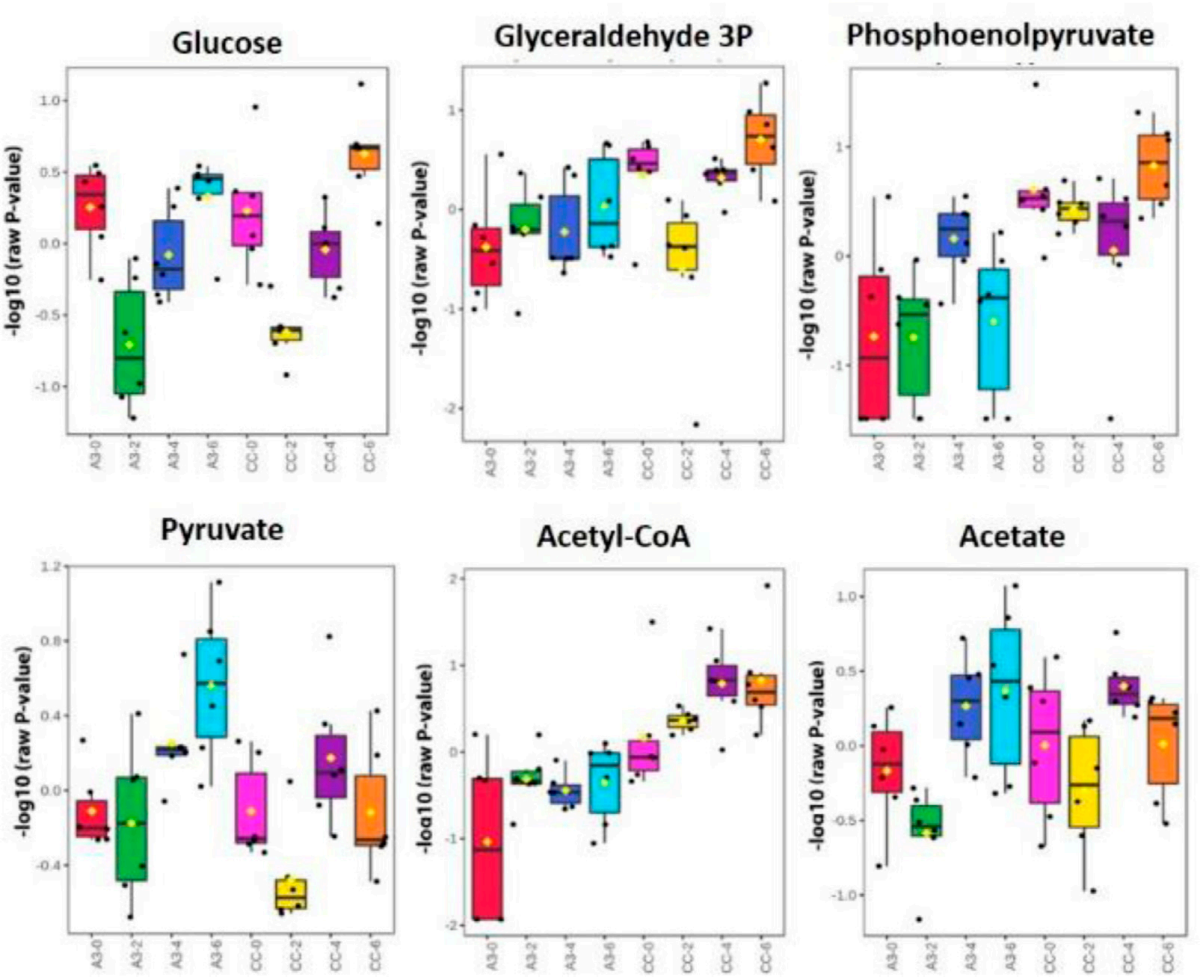
Significantly different metabolite changes in the glycolysis and gluconeogenesis pathway happening at different time points in both treated A3 (Hb5d/e3) and non-treated cells (CC) over time (0, 2, 4, and 6 h).

To further investigate this possibility, where possible, metabolites corresponding to glycolysis/gluconeogenesis were identified with changes over time, and treatments were compared using box and whisker plots (Figure 5). In Figure 5, the comparisons were made between A3-treated and control (CC, untreated MRSA) samples. These indicated some changes occurring over timelines that were treated with the established antibiotics, suggesting the value of our following changes over time. Such progressive changes were particularly noticeable with acetyl-CoA with increased levels in CC not being seen in the A3-treated samples. Considering only the samples obtained at 6 h, significant (*p* < 0.001) differences between treatments were seen with glyceraldehyde-3-phosphate, phosphoenolpyruvate, and acetyl-CoA.

## Discussion

The rise of AMR and the discovery void in the derivation of novel antibiotics has led to a revisiting of traditional medicine as sources of natural products which could represent drug leads. China has been using TCM herbs for centuries, and these are now attracting global interest as a source of natural products. However, the information regarding their use is often inaccessible to the wider scientific community. In this collaborative study, we engaged in a screening of potential antimicrobial properties and the targeting of antimicrobial metabolites from *D. crassirhizoma.*

The precise identification of the natural product obtained is a very critical step for successful innovative drug discovery. The complex chemical composition of plants or TCM material and variations in the source can lead to batch-to-batch inconsistency. Genomic techniques such as DNA barcoding are established techniques that rely on sequence diversity in short, standard *rbcL* regions (400–800 bp, and internal transcribed spacer for species-level identification (Ganie et al., 2015). Thus, we have incorporated a quality assessment based on DNA barcoding.

*D. crassirhizoma* exhibited particularly potent activity against MRSA. Sequential bioactivity-guided fractionations lead to the identification of two phloroglucinol metabolites: norflavaspidic acid AB and flavaspidic acid AB. The close similarities in their chemical structures (Figure 2) prevented them from being separated with the use of preparative HPLC or TLC. However, the considerable predominance of norflavaspidic acid AB in the most active fraction (A3) implies that this had greater anti-MRSA properties compared to flavaspidic acid AB. Norflavaspidic acid AB is an example of acyl phloroglucinol dimers formed by a methylene linkage. In terms of reported bioactivities, no antimicrobial activity has been previously reported, but norflavaspidic acid AB has inhibitory effects on melanin production by melanoma B16F10 cells with IC_50_ values of 181.3 μM (Pham et al., 2017) and against human leukemia Reh cells with IC_50_ values of 32.2 μg/ml (Ren et al., 2016). Furthermore, it countered the effects of the pro-apoptotic factor—FAS—of IC_50_ 29.7 µM (Na et al., 2006). However, we observed no cytotoxic effects for A3 against HepG2 cells where norflavaspidic acid AB was predominant. Flavaspidic acid AB has been reported to have antibacterial activity based on paper disc diffusion assays with MICs ranging between 12–20 μg/ml depending on the microorganism being tested (Lee et al., 2009) which is in line with our observations. Moreover, flavaspidic acid AB exhibited potent antioxidant activity against the LPO inhibitory test with IC_50_ values of 13.1 mM (S. M. Lee et al., 2003). It can induce IFN-α, IFN-β, and IL1-β expression in porcine alveolar macrophages, which could contribute to the inhibition of porcine reproductive and respiratory syndrome virus (PRRSV) replication (Yang et al., 2013). It also shows inhibitory effects against human leukemia Reh cells with IC_50_ values of 35.3 μg/ml (Ren et al., 2016). Its activity against the pro-apoptotic ligand FAS had an IC_50_ value of 28.7 µM (Na et al., 2006). It is now important to derive pure norflavaspidic acid AB and flavaspidic acid AB so that the anti-microbial properties of each can be properly assessed. This could be important given that we could find no evidence of cytotoxicity with these metabolites and the potencies against a clinically relevant MRSA strain.

To provide more information on how norflavaspidic acid AB/flavaspidic acid AB acts, we used metabolomic assessments in comparison to the established antibiotics based on their different modes of action. There are a series of known anti-microbial targets in MRSA, and these include cell wall inhibitors. Anti-MRSA β-lactam antibiotics include ceftobiprole and ceftaroline. Ceftobiprole is the first molecule from a new class of cephalosporins that was developed to specifically bind to mutant penicillin-binding proteins (PBPs) in MRSA (Hamad, 2010) and resists the action of class A (TEM-1) and class C (AmpC) β-lactamases (Queenan et al., 2007) and is presently in phase 3 clinical trials (Bassetti and Righi, 2015). However, rapid emergence of methicillin-resistant *S. aureus* (MRSA) containing modified penicillin-binding protein (PBP) 2a encoding operon was poorly inhibited by β-lactams (Grundmann et al., 2006). This is overcome by the glycopeptide vancomycin, which has been the drug of choice for MRSA treatment for numerous years. Vancomycin acts by inhibiting proper cell wall synthesis but almost exclusively in Gram-positive bacteria. However, because of limited tissue distribution, as well as the emergence of isolates with reduced susceptibility and *in vitro* resistance to vancomycin, the need for alternative therapies that target MRSA has become apparent (Bassetti and Righi, 2015; Hidayat et al., 2006; Micek, 2007). Alternatives can include aminoglycosides—for example, gentamycin—that block bacterial protein synthesis by inducing codon misreading and interrupting translocation of the complex between tRNA and mRNA, interacting directly with the 30S rRNA (Carter et al., 2000). Other options are DNA gyrase and topoisomerase inhibitors, which include levofloxacin and nalidixic acid. New-generation quinolones such as delafloxacin and finafloxacin show excellent results against *S. aureus* strains, especially MRSA (Kocsis et al., 2021).

Our metabolomic comparison with A3 included antibiotics with different types of anti-MRSA activities. These appeared to have similar effects at 6 h following treatment at concentrations sufficient to suppress MRSA growth by 50%. Given the different modes of action, this is suggestive of revealing a common effect such as the slowing of growth and/or associated bacterial stress. However, the A3 effect was strikingly different, suggesting a different mechanism of action to the other established antibiotics. When identifying the key metabolomic differences with A3 treatment, these appeared to include altered bioenergetic mechanisms, particularly associated with the perturbation of glycolysis/gluconeogenesis. Other metabolomic changes were observed, but these could not be clearly assigned to given pathways. Although our assessments could not identify the exact targets, for example, an enzyme, which could be inhibited by A3, the consequences appeared lead to a collapse in the levels of acetyl-CoA. Acetyl-CoA due to its importance in metabolism has been suggested to be one of a series of key “sentinel” metabolites that indicate the metabolic state of the cell (Shi and Tu, 2015). The targeting of glycolysis by A3 would explain the loss in acetyl-CoA as this provides the pyruvate which can be oxidative-decarboxylated to produce acetyl-CoA. In mammalian systems, the depletion of acetyl-CoA can lead to autophagy (Mariño et al., 2014), and in *Mycobacterium tuberculosis* perturbation of acetyl-CoA metabolism was clearly indicated to be a killing mechanism (Beites et al., 2021). Following is condensation with oxaloacetate forming citrate which can be fed into the TCA cycle, and it was surprising that this was not apparently significantly altered in our study, although TCA intermediates are relatively easy to detect using our metabolomic technology. Interestingly, flavaspidic acids have been suggested to affect cellular respiration and oxidative phosphorylation in isolated hepatocytes and, even at moderate concentrations, uncouple oxidative phosphorylation in isolated hepatocytes (Burnett et al., 1979). This is in agreement with our observations of anti-MRSA activities, but such a cross-kingdom effect would not agree with the lack of cytotoxicity against HepG2 cells*.*

## Materials and methods

### Plant materials

The commercially sourced dried plant material was provided by the Artemisinin Research Center, Institute of Chinese Materia Medica, China Academy of Chinese Medical Sciences, Beijing, China.

### DNA barcoding of plant samples

Total genomic DNA was extracted using a DNeasy Plant Mini Kit (Qiagen, United Kingdom) in accordance with the manufacturer’s instructions. The *rbcL* sequences were amplified with *rbcL*a-F: 5′-ATG​TCA​CCA​CAA​ACA​GAG​ACT​AAA​GC-3′ and *rbcL*a-R: 5′-GTAAAATCAAGTCCACCRCG-3′; DNA barcoding primers (Hollingsworth, 2011). PCR used a total volume of 20 μL with 10 μL BioMix (BioLine, United Kingdom), 1 μL of each primer (10 μM) and 1 μL of the DNA template (50 ng/µL) and 7 μL distilled water. PCR involved 1 cycle (94°C for 3 min), 35 cycles (94°C for 1 min, 55°C for 1 min, and 72°C for 1 min), and 1 cycle (72°C for 7 min). The resulting 600 bp band was sequenced using a 3730xl DNA Analyzer (Applied Biosystems, United Kingdom), with the same primers used for PCR. The derived sequences were assessed using BLAST (https://blast.ncbi.nlm.nih.gov/Blast.cgi). The *rbcL* sequence was submitted to GenBank (accession number: MN431197).

### Sequence of bioassay-guided purification

A measure of 800 g of dried material of *D. crassirhizoma* was ground into a powder and was extracted sequentially using *n*-hexane, dichloromethane (DCM), and methanol (MeOH) at room temperature with continuous stirring for 24 h, respectively. Bioactivity-linked fractionation of the *n*-hexane extract was based on the ability to suppress the growth of MRSA.

Approximately 94 g of *n-*hexane extract of *D. crassirhizoma* was fractionated using silica gel column chromatography (CC) (4 × 50 cm, 150 g of SiO_2_). The column was eluted with *n-*hexane–EtOAc (1:0 to 0:1, at 10% gradient, 500 × 4 ml of each eluent) and EtOAc–MeOH (1:0 to 1:2, 500 ml × 3 of each eluent) to yield 16 fractions (designated H for “*n*-hexane” and A through to P; thus, HA through to HP). Fraction HB (59.11 g) was again subjected to silica CC (4 × 50 cm, 150 g of SiO_2_) eluting with *n-*hexane–EtOAc (1:0 to 3:20, 1% gradient, 500 ml × 4 of each eluent) and EtOAc–MeOH (1:0 to 1:2, 5% gradient, 500 ml × 5 of each eluent) to yield 8 fractions (1 through 8; thus HB1–HB8). The HB5 fraction (10 g) was fractionated again using silica gel CC (4 × 50 cm, 150 g of SiO_2_) and eluted with *n-*hexane–EtOAc (1:0 to 1:10, 1% gradient, 500 m × 4 of each eluent), *n-*hexane–EtOAc (1:10 to 0:1, 10% gradient, 500 ml × 4 of each eluent), and EtOAc–MeOH (1:0 to 1:2, 10% gradient, 500 ml × 2 of each eluent). This resulted in 14 fractions, a through n: HB5a–HB5n.

Fractions HB5d and HB5e showed similarity in components detected by UHPLC-MS and were combined (800 mg) and further fractionated with silica CC (4 × 33 cm, 150 g of SiO_2_). Eluting with *n-*hexane–DCM (1:0 to 0:1, 10% gradient, 500 ml × 4 of each eluent) and DCM–MeOH (1:0 to 1:2, 500 ml × 4 of each eluent) yielded four fractions HB5d/e1–HB5d/e8. All the fractions were assessed by thin-layer chromatography (TLC) to check for purity.

### Bacterial growth and minimum inhibitory concentration calculation

All procedures were performed in a biosafety level 2 (BSL2) cabinet. Samples were dissolved in methanol:water (1:1) to give a final concentration of 5 mg/ml. The antimicrobial activity of all the extracts and isolated compounds was evaluated in 96-well plates by the micro-dilution technique over a concentration range (500 μg/ml to 1.5625 μg/ml^)^. Serial two-fold dilutions were performed in nutrient broth (for *Escherichia coli* ATCC 25922, *Pseudomonas aeruginosa* ATCC 27853, *S. aureus* ATCC 29213, and MRSA = methicillin-resistant *S. aureus* USA300) and nutrient broth supplemented with 0.05% Tween 80 and 0.2% glycerol (for *Mycobacterium smegmatis* NCTC 333). All diluted extracts and compounds were tested in triplicate in an initial bacterial concentration of 5.0 × 10^5^ CFU/ml. The MIC was determined as the lowest concentration of a drug at which no growth was visible at 37°C after 24 h for *E. coli*, *P. aeruginosa, S. aureus*, and MRSA and after 72 h with *M. smegmatis.* Rifampicin and gentamycin sulfate (50 μg/ml) were used as positive controls. The optical density OD_600_ was measured using a Hidex plate (Hidex Sense, United Kingdom). Growth was assessed by adding 20 μL of 0.5 mg/ml 3-(4, 5-dimethylthiazol-2-yl)-2, 5-diphenyltetrazolium bromide (MTT) stain.

### Ultra-high-performance liquid chromatography–high-resolution mass spectrometry

Fractions were analyzed on an Exactive Orbitrap mass spectrometer (Thermo Fisher Scientific), which was coupled to an Accela Ultra High-Performance Liquid Chromatography (UHPLC) system (Thermo Fisher Scientific). Chromatographic separation was performed on a reverse-phase (RP) Hypersil Gold C18 1.9 µm, 2.1 × 150 mm column (Thermo Scientific) with water using 0.1% formic acid (v/v, pH 2.74) as the mobile phase solvent A and acetonitrile:isopropanol (10:90) with 10 mM ammonium acetate as mobile phase solvent B. Each sample (20 μL) was analyzed using 0–20% gradient of B from 0.5 to 1.5 min and then to 100% in 10.5 min. After 3 min isocratic at 100% B, the column was re-equilibrated with 100% A for 7 min.

### HepG2 cell culture and MTT assay

Human Caucasian hepatocyte carcinoma (HepG2) (Sigma-Aldrich, cat# 85011430) was used for the cytotoxicity experiments. Targeted fractions were assessed for 50% of cytotoxicity (CC_50_) at concentrations from 200 to 1 μM in triplicate. Cell viability was performed using the MTT assay as described by Crusco et al. (2019).

### Standardization of antibiotic dosage

Antibiotic treatments were standardized to concentrations which inhibited growth by 50% (MIC_50_) at 6 h following microdilution of all antibiotics of a bacterial culture of OD_600_ 0.6 compared to untreated controls. The antibiotics used were chloramphenicol (CH), gentamicin (G), levofloxacin (L), nalidixic acid (N), streptomycin (S), and vancomycin (V), which were obtained from Sigma-Aldrich Ltd. (United Kingdom) and *D. crassirhizoma* metabolites.

### Sample preparation for metabolomics

The procedure previously described by Baptista et.al (2018) was followed with minor modifications. The bacteria treated with antibiotics and the untreated control group were independently cultured (*n* = 6 replicates) at constant shaking at 200 rpm at 37°C (Gallenkamp Orbital Incubator).

All samples were collected during the mid-exponential growth phase. A 3 ml aliquot of bacterial culture (OD_600_ at time 0 h was 0.6) was harvested at 0, 2, 4, and 6 h after the treatment with each respective antibiotic and control. The samples were centrifuged at 2 min, 10°C at 4500 rpm, and the pellet was resuspended with 3 ml of cold saline solution (0.85% NaCl in H_2_O w/v). The samples were stored at -80°C after the cellular metabolism was rapidly quenched with liquid N_2_ until analysis. On analysis, samples were thawed and centrifuged at 10°C at 4500 rpm for 2 min, and the pellet was washed in 4 ml of cold saline solution (0.85% NaCl in H_2_O w/v) and re-centrifuged (10°C at 4500 rpm for 2 min). All samples were adjusted to an OD_600_ of 1 and centrifuged to ensure similarly sized bacterial populations were assessed. A volume of 70 μL of the chloroform:methanol:water (1: 3: 1) mixture was added to the pellet, and extractions involved four freeze–thaw cycles with periodic vortexing. After final centrifugation at 4500 rpm, 60 μL of the particle-free supernatant was transferred into a microcentrifuge tube. An additional extraction with 50 μL of chloroform:methanol:water (1: 3: 1) was carried out, and the new supernatant, after final centrifugation, was combined with the supernatant from the first extraction. From this mixture, 50 μL was transferred into an HPLC vial containing a 0.2-ml flat-bottomed micro-insert for FIE-HRMS analysis.

### Metabolomic analyses

Extracted metabolites were analyzed by flow-infusion electrospray ionization high-resolution mass spectrometry (FIE-HRMS) in the High-Resolution Metabolomics Laboratory (HRML), Aberystwyth University. Metabolite fingerprints were created in both positive and negative ionization modes. Ion intensities were acquired between *m/z* 55 and 1200 for 3.5 min in the profiling mode at a resolution setting of 280,000 (at *m/z* 200) resulting in 3 (±1) ppm mass accuracy. Samples (20 μL volume) were injected using an autosampler into a flow of 100 μL/min methanol/water (70:30, v/v). Electrospray ionization (ESI) source parameters were set based on the manufacturer’s recommendations. An in-house data aligning routine in MATLAB (R2013b, MathWorks) was used to join mass spectra around the apex of the infusion maximum into a single mean intensity matrix (runs × *m/z*) for each ionization mode and normalized to total ion count. The derived matrices are shown in Supplementary Table S1 (https://zenodo.org/record/7111606#.YzCBjHbMKUk). Data were log_10_-transformed and used for statistical analysis performed by MetaboAnalyst 4.0 (Chong et al., 2018), which was also used to perform principal component analysis (PCA) and heat maps. The MetaboAnalyst 4.0—MS Peaks to Pathways module was used to identify metabolites (tolerance = 3 ppm) and significantly affected metabolic pathways (model organism = *S. aureus*) (Chong et al., 2018). Pathway enrichment assessments used the *mummichog* algorithm based on mass spectrometry data, avoiding the *a priori* identification of metabolites (Li et al., 2013). Mummichog plots all possible matches in the metabolic network and then looks for local enrichment, providing reproduction of true activity, as the false matches will distribute randomly (Li et al., 2013).

## Conclusion

The study also attempts to characterize the bioactives in *D. crassirhizoma* responsible for the anti-MRSA activity. Norflavaspidic acid AB and flavaspidic acid AB were identified as the major components of the fractions. The mode of action of A3 was further explored using metabolomics. Although it is currently not unequivocally established how A3 causes the observed MRSA cell death, our metabolomic approach suggested some potential mechanisms. Whether one or more of these mechanisms are the main effects or how far they are to the so-called off-target effects requires further work. However, this study demonstrated that the phloroglucinol derivatives in *D crassirhizoma* including norflavaspidic acid AB and flavaspidic acid AB have potent antibacterial activity against MRSA. One or both compounds may be a new class of anti-MRSA antibiotics. Taken together, we have demonstrated how bioactives in TCM can be targeted and purified and, using metabolomic approaches, a likely mode of action can be suggested. This approach would be extended more widely to define drug leads in TCM.

## Data Availability

The datasets presented in this study can be found in online repositories. The names of the repository/repositories and accession number(s) can be found at: NCBI GenBank, MN431197

## Supplementary material

Supplementary Table S1
*m/z* matrix derived following flow-infusion electrospray mass spectrometry. https://zenodo.org/record/7111606#.YzCBjHbMKUk
