# Corrigendum: Identification and metabolomic characterization of potent anti-MRSA phloroglucinol derivatives from *Dryopteris crassi rhizoma* Nakai (Polypodiaceae)

**DOI:** 10.3389/fphar.2023.1235626

**Published:** 2023-06-14

**Authors:** Sumana Bhowmick, Manfred Beckmann, Jianying Shen, Luis A. J. Mur

**Affiliations:** ^1^ Department of Neurology, Stanford University School of Medicine, Stanford, CA, United States; ^2^ Institute of Biological, Environmental and Rural Studies, Aberystwyth University, Aberystwyth, United Kingdom; ^3^ Artemisinin Research Center, Institute of Chinese Materia Medica, China Academy of Chinese Medical Sciences, Beijing, China

**Keywords:** natural products, phloroglucinols, *Dryopteris crassirhizoma*, metabolomics, methicillin-resistant, *Staphylococcus aureus*

In the published article, there was an error in the **Funding** statement. The original statement, “SB was supported by an AberDoc Ph.D. scholarship from Aberystwyth University, United Kingdom,” did not include the funding granted to author JS. The correct Funding statement appears below.

## Funding

“SB was supported by an AberDoc Ph.D. scholarship from Aberystwyth University, United Kingdom. JS was supported by CACMS Innovation Fund (#CI 2021A05110) from China Academy of Chinese Medical Sciences, Beijing, China.”

The authors apologize for this error and state that this does not change the scientific conclusions of the article in any way. The original article has been updated.

